# Determining the Relationship Between Work Stress and Job Performance: A Cross-Sectional Study Among Healthcare Workers

**DOI:** 10.1155/jonm/5051149

**Published:** 2025-04-08

**Authors:** Hakan Oğuz Ari

**Affiliations:** Gülhane Faculty of Health Sciences, Department of Health Management, University of Health Sciences, Ankara, Türkiye

**Keywords:** healthcare workers, job performance, nurses, work stress

## Abstract

**Background:** Work stress and job performance are critical factors for increasing productivity and ensuring sustainability in healthcare institutions.

**Aim:** This study investigates the work stress and job performance levels of healthcare workers in Türkiye and the relationship between them.

**Methods:** The study included healthcare workers of a private hospital in Ankara, Türkiye. Data were collected using the General Work Stress Scale and the Job Performance Scale.

**Results:** In the study where 47.3% of the participants were nurses, it was observed that healthcare workers had low work stress levels and high job performance levels. Nurses had higher work stress than others but lower job performance. It was determined that there was a negative, weak and statistically significant relationship between work stress and job performance.

**Conclusion:** In order to reduce work stress and increase job performance, it is important to develop managerial intervention programs by considering variables such as healthcare workers' age, marital status, years employed in the profession and their occupations. This study provides evidence-based clues for actions that will help hospital and nursing service managers control job stress and increase clinical services' and hospitals' performance.

## 1. Introduction

Work-related stress and job performance are critical factors influencing the efficiency and long-term sustainability of organisations. In the healthcare sector, providing high-quality care, particularly by nurses and other healthcare professionals, requires optimising their job performance. When employees are able to effectively manage work-related stress, it can serve as a source of motivation, leading to increased productivity, creativity and overall performance. However, when stress surpasses manageable levels, it can have a detrimental impact on performance [1, 2].

The healthcare sector is inherently challenging and presents numerous difficulties that can negatively affect the physical and mental health of its workers. Healthcare professionals are responsible for patient care, managing emergencies, operating complex systems, providing end-of-life care and using advanced medical technology. As a result, healthcare roles, particularly nursing, are frequently classified among the most stressful professions [3–5]. Stress, especially when employees lack effective coping mechanisms, has significant psychological and physical effects. The negative consequences of work-related stress are far-reaching, affecting both individuals and organisations. Furthermore, work-related stress can decrease organisational commitment, job satisfaction and quality of care, which ultimately impacts overall organisational effectiveness. Additionally, it may contribute to absenteeism, delays in care and reduced performance levels [6, 7].

Job performance is essential for the efficient, effective and high-quality delivery of healthcare services. It refers to the extent to which employees successfully complete their job responsibilities [8, 9]. Nurses and healthcare professionals who perform their duties effectively tend to experience higher motivation, job satisfaction and a sense of self-efficacy. However, excessive work stress can reduce performance, leading to lower job satisfaction and a decline in the quality of care provided [10].

Job performance can be evaluated in two key dimensions: task performance and contextual performance. Task performance refers to behaviours that directly contribute to achieving the goals outlined in job descriptions, while contextual performance includes positive behaviours that are valued by the organisation, even if they are not explicitly outlined in the job description [11].

The job performance of healthcare workers is critical to the success of healthcare organisations. The performance of healthcare personnel directly influences the quality of care, patient outcomes and the achievement of organisational goals. Job performance is influenced not only by employees' perceptions, values and attitudes but also by their ability to apply skills, demonstrate individual talents and exert effort in fulfilling job responsibilities [12, 13].

High job performance is associated with improved service quality, better patient care and overall organisational productivity. On the other hand, poor job performance can jeopardise patient well-being and increase morbidity and mortality rates. Like any other organisation, hospitals rely on effective performance management to succeed. Therefore, evaluating and reporting the performance of healthcare workers is essential to maintaining high service quality standards. While work-related stress can have positive effects when managed effectively, excessive stress can overwhelm an individual's ability to cope, leading to harmful outcomes [12, 14].

A review of the literature on the relationship between work-related stress and job performance reveals mixed results. Some studies indicate that high stress levels decrease job performance [15–22], while others suggest that moderate stress may enhance performance in certain contexts [23–25]. Additionally, some research shows that employees with moderate levels of stress tend to perform better than those experiencing either high or low stress levels [26, 27].

The inconsistency in these findings highlights the need for further investigation into the relationship between work stress and job performance. This relationship is a critical area that requires careful attention, particularly for health and hospital administrators. The primary aim of this study is to explore how work stress affects job performance among nurses and other healthcare professionals and to gain a better understanding of this dynamic.

## 2. Materials and Methods

### 2.1. Design

This descriptive, cross-sectional study was conducted to investigate the relationship between work-related stress and job performance among healthcare workers.

### 2.2. Study Sample

The study was conducted with healthcare professionals (nurses, laboratory technicians, radiology technicians, anaesthesia technicians, dietitians, opticians, biologists and physiotherapists) employed at a private hospital in Türkiye. Physicians were excluded from the study, as the hospital administration only granted research permission to nonphysician personnel. The data collection took place between October and November 2024.

The inclusion criteria for the study were working at the hospital where the research was conducted for at least six months and being a nonphysician healthcare professional. The exclusion criteria were working at the hospital for fewer than six months.

A total of 230 healthcare professionals were employed at the private hospital where the study was conducted. In this study, no specific sample size was determined, as the aim was to reach the entire population. Data were collected from 207 healthcare professionals (90% of the population) through a survey. Nurses (*n* = 98) represented the largest group within the population. The questionnaires were administered to the participants using the face-to-face interview technique by the researcher, and the data were subsequently obtained.

### 2.3. Data Collection Method

Prior to conducting the research, the necessary institutional permissions and ethical approval were obtained. The survey was conducted using printed paper questionnaires and administered face-to-face during a specific time window to avoid overlapping with healthcare workers' shifts and to ensure accurate responses. This window coincided with the hospital's annual in-service training sessions. With hospital management approval, surveys were administered immediately after the training to groups of 35–40 individuals in the training room. Participants were provided with the necessary information and gave their consent before completing the surveys. The completed surveys were then collected and stored by the researcher.

### 2.4. Data Collection Instruments

#### 2.4.1. Personal Information Form

The demographic and occupational profiles of the participants were assessed using a set of 8 questions, including items related to age, gender, professional experience, marital status, occupation and other relevant factors. These questions were designed to gather information that would support the study's objectives.

#### 2.4.2. General Work Stress Scale (GWSS)

The GWSS, developed by De Bruin [28] and adapted for Turkish validity and reliability by Teles [29], was used to assess the level of work-related stress perceived by the participants. The GWSS is a 5-point Likert-type scale, consisting of a single dimension and 9 items (1 = *Never*, 5 = *Always*). The overall Cronbach's alpha coefficient for the scale was 0.91, and the Spearman–Brown reliability coefficient was 0.89.

#### 2.4.3. Job Performance Scale (JPS)

To measure the job performance of the participants, the JPS developed by Çalışkan and Köroğlu [30] was utilised. The JPS is a 5-point Likert scale consisting of two subdimensions: task performance and contextual performance, with a total of 11 items (1 = *Strongly disagree*, 5 = *Strongly agree*). The reliability analysis revealed that the scale demonstrated high reliability, with an overall Cronbach's alpha coefficient of 0.914.

### 2.5. Ethical Approval

This research received approval from the Social Sciences and Humanities Ethics Committee of OSTIM Technical University (09.10.2024, E-96274976-100-37236). Data collection commenced following the work permit granted by the institution where the research was conducted, dated 19.08.2024 and numbered E.00000003-020-8. The entire study was designed and carried out in accordance with the principles outlined in the Declaration of Helsinki. All participants were informed about the study's objectives and were assured that they could withdraw from the interview at any time. Furthermore, participants' rights were protected through voluntary consent forms, and their consent was obtained prior to participation.

### 2.6. Statistical Analysis

The data were analysed using SPSS 25.0 software. Descriptive statistics, including frequency, percentage, arithmetic mean, minimum, maximum and standard deviation, were calculated. To assess the normality of the data distribution, the Shapiro–Wilk test, along with kurtosis and skewness values, were examined. For data that were not normally distributed, nonparametric tests were applied. The Mann–Whitney *U* test and Kruskal–Wallis test were used to assess differences based on sociodemographic characteristics and variables. Cronbach's alpha coefficient was calculated to determine the measurement reliability of the scales, while the relationship between variables was evaluated using Spearman's Rho correlation coefficient. A statistical significance level of *p* < 0.05 was considered for all analyses.

## 3. Results

The average age of the participants was 30.8 ± 8.02 years, with 47.3% of them being nurses. The average total years of experience in their profession was 6.4 ± 6.3 years, while the average duration of employment at the current hospital was 4.7 ± 4.9 years. Additionally, the average length of time spent in their current department was 4.3 ± 4.6 years. Of the participants, 85% were women, 56.5% held an associate degree, and 52.2% were married (see Table 1).

The average score of the participants on the GWSS was 1.95 ± 0.68. A lower score on the scale, or a lower overall average, indicates a low level of work stress, while a higher score suggests a high level of work stress. The lowest score recorded by the participants was 1, and the highest score was 4.22. Based on these results, it can be concluded that the participants' work stress levels are generally low. Cronbach's alpha coefficient, which measures the overall reliability of the scale, was calculated to be 0.891, indicating that the scale is highly reliable (see Table 2).

The average score of the participants on the JPS was 4.38 ± 0.76. When examining the subdimensions of the scale, participants scored an average of 4.38 ± 0.83 on the task performance subdimension and 4.38 ± 0.76 on the contextual performance subdimension. High average scores in both the scale and its subdimensions indicate that the participants' job performance levels are generally high. Based on these results, it can be concluded that, on average, the participants' evaluations of their job performance are high. Cronbach's alpha coefficient, which reflects the overall reliability of the scale, was calculated to be 0.963, indicating that the scale is highly reliable (see Table 2).

Statistically significant differences were found in the participants' age, marital status, total working time in the profession and occupation with regard to the average total scores obtained from the GWSS. Further analysis revealed that participants aged 30 and under had significantly lower scores (mean score: 2.13 [SD 0.72]) compared to those in the 31–40 (mean score: 1.76 [SD 0.54]) and over 41 age (mean score: 1.62 [SD 0.55]) groups. Additionally, married participants scored significantly higher (mean score: 2.06 [SD 0.72]) than their single counterparts (mean score: 1.84 [SD 0.64]), those with 5 years or less of total working experience in the profession had higher scores (mean score: 2.04 [SD 0.74]) compared to those with 6–10 years of experience (mean score: 1.71 [SD 0.56]), and nurses had significantly higher scores (mean score: 2.38 [SD 0.64]) than other healthcare workers (mean score: 1.55 [SD 0.44]). No significant differences were found in terms of gender, educational status or working hours in the hospital and department (see Table 3). A low score on the items or overall scale of the GWSS indicates a low level of work-related stress, while a high score indicates a high level of work-related stress.

Regarding the average total scores from the JPS, statistically significant differences were observed between occupations. Specifically, nurses had significantly lower average scores (mean score: 4.28 [SD 0.80]) on the JPS compared to other healthcare professionals (mean score: 4.47 [SD 0.72]). No significant differences were found for JPS mean total scores based on other variables (see Table 3). High average scores from the overall JPS scale and its subdimensions indicate a high level of job performance, while low scores indicate a low level of job performance among participants.

When the relationship between the GWSS and the JPS, along with its subdimensions, was examined, a negative, weak and statistically significant correlation was found between the GWSS and the JPS (*r* = −0.211). Additionally, strong and very strong statistically significant relationships were observed between the JPS and its subdimensions. This suggests that an increase in work stress levels among nurses and other healthcare professionals negatively impacts their job performance. However, it is important to note that this relationship is weak (see Table 4).

Based on the results of the correlation analysis, performing regression analysis to examine the causal relationships was deemed inappropriate due to the weak association between the variables, as well as the failure to meet the critical assumptions of normality and linearity required for regression analysis.

## 4. Discussion

This study aimed to assess the general work stress and job performance levels of healthcare workers and explore the relationship between these two factors. The findings indicate that, overall, healthcare workers report low levels of work stress, which aligns with the results of Ribeiro et al. [31]. Despite facing high stress loads, healthcare workers are exposed to various stressors such as the uncertainty surrounding medical procedures, the need for rapid responses to patients and their families and the significant consequences of medical errors. Furthermore, long working hours, night shifts and understaffing contribute to increased stress levels in healthcare settings [32]. When examining the reasons for the generally low levels of work-related stress reported by the participants, it appears that this is closely related to sociodemographic characteristics. Specifically, participants aged 30 and above, unmarried individuals, those with 5 or more years of professional experience and healthcare workers other than nurses reported very low levels of work-related stress, and these findings were also statistically significant.

In terms of job performance, the study reveals that healthcare workers generally rate their performance as above average, in both the task performance and contextual performance subdimensions, as well as at the overall level. This finding is consistent with the results of Deng et al. [33], who reported high job performance levels across all subdimensions and the overall scale in their study of healthcare workers. In recent years, job performance has become a key focus for organisations, particularly in healthcare. Performance is primarily related to processes that emphasise employee behaviours and the outcomes resulting from these behaviours. In other words, job performance consists of measurable actions, behaviours and results that contribute to the organisational goals and values [34–36].

The findings of this study indicate that demographic factors significantly influence healthcare workers' experiences with work stress and job performance. Specifically, age, marital status, years of experience in the profession and occupation have notable effects on these experiences.

Given that the findings related to nurses represent a unique case, a more specific evaluation is warranted. Nurses experience higher levels of work stress compared to other occupational groups, which in turn negatively impacts their job performance. Analysis of the GWSS scores reveals that nurses report significantly higher stress levels than other healthcare professionals, while their JPS scores are lower. These results align with existing literature, where similar findings have been reported. For instance, in Abualrub's study [37], it was found that nurses with lower stress levels exhibited higher job performance. The study emphasised the need for stress reduction programs to enhance the work performance of nurses [38]. Additional research on the relationship between work stress and job performance also highlights that nurses experience more stress than other healthcare workers, with stress levels among nurses being notably higher than those of other healthcare professions [39–42].

Younger healthcare professionals (aged 30 and under) experience higher levels of stress compared to their older counterparts. Consistent with the findings of the present study, it is evident that stress levels tend to decrease as healthcare workers age. This suggests that the relatively lower levels of experience among younger workers may contribute to the heightened stress they encounter in the workplace [43–45].

Marital status, another sociodemographic variable with a significant impact on stress levels, was also found to influence stress. Specifically, married individuals reported higher stress levels than their single colleagues. This finding is supported by existing literature, which indicates that married individuals often face greater work-related stress than their unmarried counterparts. The dual responsibility of managing both professional and familial obligations may place married employees under more pressure, contributing to their elevated stress levels [46, 47].

In alignment with studies by Qosaj and Spendelow [44, 48], the current study also found that employees in the early stages of their careers experienced higher stress levels compared to their more experienced peers. These results suggest that the length of time spent in the profession plays a significant role in shaping stress levels, with stress generally decreasing as years of professional experience accumulate.

The study found that job performance varied significantly across different healthcare professions. Specifically, nurses reported significantly lower average scores on the JPS compared to other healthcare professionals. This disparity can primarily be attributed to the higher levels of work stress experienced by nurses relative to other healthcare workers. However, no significant differences in job performance were observed when analysed according to other sociodemographic variables.

The most significant finding of this study is the statistically significant, negative and weak correlation between work stress and job performance among healthcare workers. The results suggest that higher levels of work stress have a detrimental effect on job performance. This finding is consistent with a study by Tekingündüz et al. [18], which also identified a negative relationship between work stress and job performance in healthcare workers. Furthermore, Izadi et al. research [49] on work stress revealed significant negative correlations between employee performance and work stress. In addition, Alayoubi et al. [50], in their study on the relationship between job performance and work stress, found that nurses are among the occupational groups most affected by stress, with job performance declining as stress levels increase.

In conclusion, this research highlights key areas of focus for healthcare and nursing services managers who aim to ensure effective, efficient and high-quality service delivery within healthcare institutions. In particular, it underscores the importance of addressing employee stress levels to enhance institutional performance. As is common in all organisations, work stress represents a prevalent and costly challenge within hospitals. Healthcare workers, particularly nurses, face significant stress due to the high level of responsibility, the expectations placed upon them and the lack of sufficient authority. Work stress is a critical factor that undermines hospital effectiveness and efficiency, while also negatively impacting the quality of care provided to patients [51–53].

From a healthcare perspective, the fundamental assumption underlying accessible and affordable healthcare delivery is that health service providers must continuously improve their performance and ensure its sustainability. Given that the core activity of hospitals involves patient care and treatment, it is evident that the provision of quality healthcare services is not achievable without the active participation of all institutional personnel [22, 54].

### 4.1. Limitations and Future Research

The findings of this study are limited to the responses of healthcare workers at a private hospital in Ankara, Türkiye, and therefore cannot be generalised to all healthcare workers or the broader population. Additionally, it is important to acknowledge the potential for response bias among participants, which may affect the validity of the results. These limitations should be carefully considered when interpreting the findings.

Future research would benefit from examining healthcare workers in both private and public hospitals, as this would provide a more comprehensive understanding of the relationships between work stress and job performance. Furthermore, employing diverse context-specific measurement tools and scales could reveal new insights and offer a more nuanced evaluation of the concepts of work stress and job performance, as well as the dynamics between them.

## 5. Conclusion

This cross-sectional, single-centre study offers valuable insights into the relationship between work stress and job performance among healthcare workers. As work stress, particularly among nurses, increases, there is a corresponding decline in job performance, which may serve as a foundation for future studies that explore the effectiveness of hospitals and healthcare systems. By highlighting the connections between work stress and job performance, the study also emphasises the importance of controlling variables such as age, marital status, years of experience and occupation, all of which play a significant role in this context. The findings provide new evidence for interventions aimed at improving institutional performance and the development of targeted policies. Additionally, this study lays the groundwork for future research and practical applications in this area.

## Figures and Tables

**Table 1:** Sociodemographic and occupational characteristics of the participants (*N* = 207).

	*N*	%	Mean ± SD
Profession			
Nurse	98	47.3	
Other^∗^	109	52.7	
Age (years)			30.8 ± 8.02
30 ≤	117	56.5	
31–40	60	29	
≥ 41	30	14.5	
Gender			
Female	176	85	
Male	31	15	
Education			
High school	40	19.3	
Associate degree	117	56.5	
Undergraduate and above	50	24.2	
Marital status			
Single	99	47.8	
Married	108	52.2	
Years employed in the profession			6.4 ± 6.3
≤ 5	126	60.9	
6–10	38	18.4	
≥ 11	43	20.8	
Years employed in the hospital			4.7 ± 4.9
0–2	93	44.9	
3–5	58	28	
6+	56	27.1	
Years employed in the department			4.3 ± 4.6
0–2	103	49.8	
3–5	54	26.1	
6+	50	24.2	

^∗^Laboratory technicians, radiology technicians, anaesthesia technicians, dietitians, opticians, biologists and physiotherapists.

**Table 2:** Average scores obtained from scales and reliability of scales.

Scale	*N*	Mean	No. of items	Std. deviation	Minimum	Maximum	Cronbach's alpha
GWSS	207	1.95	9	0.68	1.00	4.22	0.891
JPS	207	4.38	11	0.76	1.27	5.00	0.963
Task performance	207	4.38	5	0.83	1.40	5.00	0.951
Contextual performance	207	4.38	6	0.76	1.00	5.00	0.938

*Note:* The minimum score that can be obtained from the scale is 1, and the maximum score is 5.

**Table 3:** Comparison of scale scores according to sociodemographic characteristics.

Demographic data/scales		*n*	GWSS				JPS			
			$\overset{¯}{x}\pm \text{sd}$	*t*	*p*	Difference	$\overset{¯}{x}\pm \text{sd}$	*t*	*p*	Difference
Age	≤ 30^1^	117	2.13 ± 0.72	20.01	**0.01**	**1 > 2** **1 > 3**	4.45 ± 0.69	4.42	0.11	
	31–40^2^	60	1.76 ± 0.54				4.17 ± 0.97			
	≥ 41^3^	30	1.62 ± 0.55				4.54 ± 0.41			

Gender	Female^1^	176	1.97 ± 0.72	0.12	0.73		4.38 ± 0.78	0.35	0.55	
	Male^2^	31	1.83 ± 0.41				4.37 ± 0.65			

Education	High school^1^	40	1.90 ± 0.60	3.87	0.14		4.13 ± 1.01	5.23	0.07	
	Associate degree^2^	117	1.88 ± 0.67				4.46 ± 0.70			
	Undergraduate and above^3^	50	2.12 ± 0.76				4.40 ± 0.60			

Marital status	Single^1^	99	1.84 ± 0.64	5.23	**0.02**	**2 > 1**	4.38 ± 0.76	0.08	0.77	
	Married^2^	108	2.06 ± 0.72				4.38 ± 0.75			

Years employed in the profession	≤ 5^1^	126	2.04 ± 0.74	10.15	**0.04**	**1 < 4**	4.34 ± 0.81	4.89	0.09	
	6–10^2^	38	1.71 ± 0.56				4.66 ± 0.32			
	≥ 11^3^	43	1.88 ± 0.54				4.25 ± 0.83			

Years employed in the hospital	0–2^1^	93	1.88 ± 0.65	6.02	0.12		4.44 ± 0.70	0.41	0.82	
	3–5^2^	58	2.15 ± 0.83				4.30 ± 0.84			
	≥ 6^3^	56	1.84 ± 0.53				4.37 ± 0.77			

Years employed in the department	0–2^1^	103	1.93 ± 0.70	6.87	0.76		4.42 ± 0.73	0.78	0.68	
	3–5^2^	54	2.09 ± 0.75				4.25 ± 0.90			
	≥ 6^3^	50	1.83 ± 0.55				4.44 ± 0.64			

Profession	Nurse^1^	98	2.38 ± 0.64	84.33	**0.01**	**1 > 2**	4.28 ± 0.80	7.34	**0.01**	**1 < 2**
	Other^2∗^	109	1.55 ± 0.44				4.47 ± 0.72			

*Note:* Bolded values denote statistically significant differences.

^∗^Laboratory technicians, radiology technicians, anaesthesia technicians, dieticians, opticians, biologists and physiotherapists.

^1,2,3^Employed to rank and differentiate between demographic variables.

**Table 4:** Correlation analysis results for the relationship between General Work Stress Scale and Job Performance Scale.

Variables	1	2	3	4
General Work Stress Scale (1)	1			
Job Performance Scale (2)	−0.211^∗∗^	1		
Task performance (3)	−0.099	0.905^∗∗^	1	
Contextual performance (4)	−0.270^∗∗^	0.930^∗∗^	0.717^∗∗^	1

^∗∗^Correlation is significant at the 0.01 level (2-tailed).

## Data Availability

The data that support the findings of this study are available from the corresponding author upon reasonable request.
